# Outcomes of MyoRing Implantation in Eyes with Keratoconus in the Eastern Province of Saudi Arabia: “A Single-Arm Cohort Study”

**DOI:** 10.1155/2019/2630704

**Published:** 2019-08-29

**Authors:** Yousef Saad Alshammari, Abdulaziz Ismail Al Somali

**Affiliations:** ^1^Division of Cornea and External Eye Disease Segment, Dhahran Eye Specialist Hospital, Dhahran, Saudi Arabia; ^2^Division of Cornea and External Eye Disease, Department of Ophthalmology, King Faisal University, Al Ahsa, Saudi Arabia

## Abstract

**Purpose:**

To evaluate the efficacy and safety of MyoRing implantation in eyes with keratoconus managed at a tertiary eye hospital in the Eastern Province of Saudi Arabia.

**Methods:**

This one-armed historical cohort study included keratoconus patients operated for MyoRing implant. The cases were assessed before and 6 months after surgery. Uncorrected visual acuity (UCVA) and best-corrected visual acuity (BCVA), spherical equivalent (SE), central corneal thickness (CCT), and corneal curvature (Kmean) were noted and measured before and 6 months after the intervention. Intra- and postoperative complications were noted.

**Result:**

We studied 12 eyes of 12 patients with moderate keratoconus. The median of Kmean was 50.6 mm (IQR 47.54; 52.5) and 44.5 mm (IQR 42.5; 46.8) before and 6 months after surgery. The change in Kmean was significant (*P*=0.002). The median spherical equivalent (SE) was −5.1D (interquartile range (IQR) 7.1; −3.6) and −0.6 (IQR −2.1; 0.8) before and 6 months after surgery. The difference in SE was significant (Wilcoxon signed-rank test *P*=0.004). The CCT was 447 ± 34 *μ*m and 444 ± 30 *μ*m before and 6 months after surgery, respectively. The CCT change was not significant (*P*=0.26). The UCVA and BCVA improved by 2 or more lines in 9 (75%) eyes, remained stable in 2 (16.7%) eyes, and decreased in 2 (16.7%) eyes.

**Conclusion:**

MyoRing implant seems to be a safe and effective procedure to manage low and moderate keratoconus. The outcomes could be further enhanced by additional procedures such as collagen cross-linkage and photorefractive keratectomy if warranted.

## 1. Introduction

Keratoconus is a progressive, asymmetric disorder associated with structural changes in the arrangement of corneal collagen [1]. Keratoconus in children is more aggressive and differs from adult keratoconus [2]. Although keratoplasty is the method of choice for treating advanced keratoconus, alternatives include contact lens, collagen cross-linking (CXL), and intrastromal ring implantation [3–7]. Combination of these methods can also be performed. Stable keratoconus and a lack of cornea from nearby eye banks often prompt surgeons to look for an alternative procedure.

The prevalence of keratoconus in Saudi Arabia is 4.8% among those 6 to 21 years old [8]. The incidence in the Asir province of Saudi Arabia was reported to be 20 per 100,000 population [9]. In the Eastern Province of Saudi Arabia, there are numerous cases of keratoconus; however, corneal donor tissue has to be transported elsewhere for corneal transplantation. In such circumstances, alternative management modalities can aid in delaying the need for keratoplasty in keratoconus until donor material is available.

In view of the limited availability of donor tissue, alternative procedures are needed to manage keratoconus. Intrastromal ring implantation for myopia has been previously documented in the Saudi population [10, 11]. A study from Riyadh, Saudi Arabia, reported promising results with a combined procedure of CXL, ring segment implantation, and refractive surgery [12]. To the best of our knowledge, there are no studies on the outcomes of MyoRing (DIOPTEX GmbH, Linz, Austria) implantation as an alternative surgical procedure to manage keratoconus in Saudi Arabia.

In this study, we present the six-month outcomes of intrastromal MyoRing implantation in eyes with keratoconus managed at a tertiary eye hospital in the Eastern Province of Saudi Arabia.

## 2. Methods and Materials

This one-armed historical cohort study was approved by the institutional research board of Dhahran Eye Specialist Hospital, Dhahran, Saudi Arabia. Patients with keratoconus scheduled for MyoRing intrastromal ring implantation between January 2017 and December 2017 were included in this study. MyoRing is a 360° continuous full-ring implant to be implanted into a corneal pocket for the treatment of myopia and keratoconus. Depending on the grade of myopia and keratoconus, the diameter of the MyoRing ranges from 5 to 8 mm and the thickness ranges from 200 to 320 microns. Written informed consent was obtained from all patients. This study adhered to the tenets of the Declaration of Helsinki 1975 as revised in Fortaleza 2013.

One corneal surgeon and one general ophthalmologist were involved in the study. Patients with moderate keratoconus (levels 2 and 3 as per Amsler–Krumeich classification) consenting to undergo intrastromal corneal ring implant for managing keratoconus were included in this study. Those using contact lens were included, but those having undergone corneal surgery in the past were excluded. Patients with other ocular comorbidities and refusing to participate in the study were excluded from the study.

The uncorrected distance visual acuity (UCVA) was measured using a Snellen chart projected at 10 feet and viewed through a mirror. If the top letter was not visible, the vision was retested at 5 feet. Pinhole acuity was used to determine the best-corrected visual acuity (BCVA). Vision in each eye was tested separately. The refraction was performed using a Spot Screener (Heine Optotechnik, Herrsching, Germany) and was documented as sphere, cylinder, and axis and BCVA.

All patients had a comprehensive eye assessment including slit-lamp biomicroscopy and dilated fundus examination. The corneal imaging was performed with the Pentacam device (OCULUS Optikgeräte GmbH, Wetzlar, Germany). Keratoconus was graded using the Amsler–Krumeich classification based on the distribution area of ectasia as previously described [13].

Data were collected on patient age, gender, and eye. In patients with bilateral keratoconus, the first eye that underwent surgery was included in the present study. The preoperative topographic data included K1 and K2 measurements and the steep axis of astigmatism. The central corneal thickness and location of the cone were also noted. The location was graded as central, paracentral overlapping pupillary area, and totally decentered.

All surgical procedures were performed under topical anesthesia by one experienced surgeon (AM). The central point of the site of MyoRing implantation (DIOPTEX) was marked under magnification with an operative microscope. A stromal pocket was created at the depth of 300 *μ*m and a diameter of 9 mm followed by a 4.5 mm wide tunnel incision superiorly using an Alcon/WaveLight FS200 femtosecond laser (Alcon Surgical, Fort Worth, TX, USA). The surgical details have been previously described. [14] Then, the MyoRing was inserted into the pocket. The manufacturer's nomogram was used to calculate the size of the MyoRing for implantation. At the end of the procedure, a bandage contact lens was placed. None of the patients required sutures. Postoperatively, all patients were prescribed topical prednisolone acetate ophthalmic suspension 1.0% (Optipred, Jamjoom Pharma, Saudi Arabia) four times a day, topical moxifloxacin ophthalmic solution 5.0% (Moxicip, Cipla, India) four times a day, and nonpreserved artificial tears four times a day. Antibiotic eye drops were discontinued at 1 week postoperatively, and steroid eye drops were tapered over 4–6 weeks. Bandage contact lenses were removed on the first postoperative day.

The data were collected using a pretested data collection form. Statistical Package for Social Sciences (SPSS 24) (IBM Corp., Armonk, NY, USA) was used for univariate analysis with a nonparametric method. The qualitative variables are presented as numbers and percentage proportions. The continuous variable was plotted to study normality curve. If distributed normally, we calculated their mean and standard deviation. If its distribution was not normal, we calculated median and interquartile range (IQR). To compare the qualitative data preoperatively and six months postoperatively, a 2 × 2 table was used and two-sided *P* values were calculated. For continuous variables, the two-sided Wilcoxon *P* value was calculated (by using the Wilcoxon signed-rank test in nonparametric tests in SPSS). *P* < 0.05 was considered statistically significant.

## 3. Results

The study sample was comprised of 12 eyes of 12 patients with moderate keratoconus. Their median age was 32 years (interquartile range 25.3; 34.6). Six (50%) patients were males, and 4 (33.3%) right eyes and 8 (66.7%) left eyes underwent surgery. The location of keratoconus cone was central in 3 (25%), paracentral in 8 (66.7%), and totally decentered in one (8.3%) eyes.

The corneal parameters and refractive status before and 6 months after MyoRing implantation were compared (Table 1). The corneal curvature improved six months after surgery compared to before MyoRing implantation. There was an improvement in corneal curvature (*P*=0.002), sphere (*P*=0.002), cylinder (*P*=0.02), and spherical equivalent refractive status (Wilcoxon *P*=0.002). However, there was no statistical change in CCT (*P*=0.6).

The change in UCVA and BCVA from preoperatively to 6 months after surgery was compared. The UCVA and BCVA improved by 2 or more lines in 9 (75%) eyes, remained stable in 2 (16.7%) eyes, and decreased in 2 (16.7%) eyes. These two eyes had decentered cone and had progression of keratoconus from moderate to severe grade. In our study, we did not find any intraoperative or postoperative complications.

## 4. Discussion

In this cohort of moderate keratoconus managed by intrastromal MyoRing implantation, we noted a stabilization of the disease in the early postoperative period. Additionally, the increased postoperative visual acuity was likely due to the improved regularity of the front corneal surface. As expected, the central corneal thickness did not change significantly. Conventionally, the combination of CXL and PRK is used with intrastromal implants for achieving good anatomic and refractive outcomes. Our study with short-term follow-up showed positive gains even with a single procedure.

Barbara et al. [15] have described the mechanism of action of the MyoRing compared to other intrastromal ring segments. MyoRing differs from other intrastromal rings for corneal ectasia and the correction of refractive error. For example, it is not segmental but placed as a full circle and is rigid with an elastic module to change the corneal curvature. The MyoRing can be inserted through a small opening into the corneal stroma without weakening the corneal architecture.

A study of the Ferrara ring concluded that 210° ring provides better visual outcomes compared to 160° ring implantation [16]. Hence, the MyoRing is a 360° ring which may result in better visual outcomes. A study of Keraring implantation for keratoconus reported greater than 90% success [6]. However, the researchers in the previous study used both PRK and CXL with intrastromal ring implantation to achieve these outcomes [6]. Hence, comparison to our study may not be appropriate. Pashtaev et al. [17] compared different types of intrastromal implants and found better outcomes with the MyoRing. They partially attributed this outcome to the 360° insertion of the MyoRing [17]. Studený et al. [18] reported good outcomes at 12 and 24 months after MyoRing implantation.

Our outcomes indicated remarkable success in decreasing corneal irregularities postoperatively. Although Mohebbi et al. [19] reported good outcomes, they also reported endothelial cell loss following MyoRing insertion. We did not study this complication because we assumed that the manipulation of the anterior surface and stroma that was peripheral to the cone is less likely to affect endothelium.

In the current study, 2 eyes with moderate keratoconus at the time of recruitment experienced a decrease in visual acuity with a decentered cone and progression postoperatively. Hence, good outcomes with the MyoRing are contingent on appropriate patient selection.

Vega-Estrada and Alio [20] found that femtosecond laser creation of the tunnel for insertion of the MyoRing has resulted in more precise placement and less damage to the corneal stroma.

In our series, there were no intraoperative or postoperative complications. A previous study of the Keraring implant reported a few cases of decentration, keratitis, and implant extrusion [6, 16]. Minimizing instrumentation and manipulation within the corneal layers by using a femtosecond laser and following strict aseptic measures likely limited the chance of postoperative infection in the current study. The advantage of the MyoRing for moderate keratoconus is that other procedures such as CXL or PRK can be performed as required.

It should be noted that MyoRing is a full ring and its behavior therefore should resemble ICRS (320 and 340) that were marketed subsequently [21].

There are some limitations to our study. As this was a one-armed cohort study, we could not compare the outcomes with other conventional treatment modalities for keratoconus. Further studies are recommended to evaluate the additive beneficial effect of the MyoRing implant for keratoconus. Our study has a small sample size, and information on different confounders was not collected and studied. Only six-month follow-up was available for this study; hence, the outcomes therefore cannot be extrapolated for longer duration and conclude us with confidence about stabilization of corneal changes. Long-term outcomes are reported for MyoRing [22]. Similar long-term outcomes of the MyoRing implant would be useful to determine the utility as a primary procedure for keratoconus. In cases of postoperative hyperopia, one may also take implantation of ICL into account. The outcomes of the current study indicate that MyoRing seems to be a safe and effective procedure to manage low and moderate keratoconus. The outcomes could be further enhanced by additional procedures such as CXL and PRK if warranted.

## Figures and Tables

**Table 1:** Corneal parameters and refractive status before and 6 months after MyoRing implantation in eyes with keratoconus.

	Before implant		Six months after MyoRing implant		Validity (Wilcoxon *P*)
	Median	IQR^*∗*^	Median	IQR	
K1	47.1	45.5; 5.0.0	43.2	40.4; 44.7	0.002
K2	53.0	49.0; 55.6	46.2	44.4; 48.8	0.003
K_mean_	50.6	47.54; 52.5	44.5	42.5; 46.8	0.002
Difference of K (K2–K1)	4.8	2.8; 5.8	4.1	2.1; 5.1	0.2
Spherical	−3.3	−4.8; −1.8	0.0	−0.75; 1.5	0.009
Cylinder	−4.0	−4.5; −2.6	−1.6	−2.7; 1.1	0.02
Spherical equivalent (SE)	−5.1	−7.1; −3.6	−0.4	−2.1; 0.75	0.004
Central corneal thickness (CCT) (*μ*m)	448.5	419.8; 470	447.5	416; 460.3	0.26

^*∗*^IQR = interquartile range.

## Data Availability

The eye, age, sex, K1, K2, UCVA, BCVA, refraction, CCT, and CONE data used to support the findings of this study are included within the supplementary information file.

## Supplementary Materials

Supplementary Materials Preoperative data include MRN (medical record number), eye, age, sex, K1 (flattest keratometry readings on Pentacam), K2 (steepest keratometry readings on Pentacam), UCVA (uncorrected visual acuity), BCVA (best-corrected visual acuity), Refract (refraction), CCT (central corneal thickness), and CONE (the location of the cone on the Pentacam). Postoperative data include MRN (medical record number), eye, age, sex, K1 (flattest keratometry readings on Pentacam), K2 (steepest keratometry readings on Pentacam), UCUCVA (uncorrected visual acuity), BCVA (best-corrected visual acuity), Refract (refraction), CCT (central corneal thickness), and CONE (the location of the cone on the Pentacam).
