# Supplementary material for: Outcomes of MyoRing Implantation in Eyes with Keratoconus in the Eastern Province of Saudi Arabia: “A Single-Arm Cohort Study”
Source: J Ophthalmol. 2019 Aug 29;2019:2630704. doi: 10.1155/2019/2630704 (PMC6735184; doi:10.1155/2019/2630704)
Supplement: Supplementary Materials — Preoperative data include MRN (medical record number), eye, age, sex, K1 (flattest keratometry readings on Pentacam), K2 (steepest keratometry readings on Pentacam), UCVA (uncorrected visual acuity), BCVA (best-corrected visual acuity), Refract (refraction), CCT (central corneal thickness), and CONE (the location of the cone on the Pentacam). Postoperative data include MRN (medical record number), eye, age, sex, K1 (flattest keratometry readings on Pentacam), K2 (steepest keratometry readings on Pentacam), UCUCVA (uncorrected visual acuity), BCVA (best-corrected visual acuity), Refract (refraction), CCT (central corneal thickness), and CONE (the location of the cone on the Pentacam). [file 2630704.f1.pdf]

NO.	MRN	Eye	Age	Sex	K1	K2	UCVA	BCVA	Refract.	CCT	Cone
1	30490	OD	25	F	45.2@13.7	48	20/200	20/50	-2.50-2.50@50	406	Asymm.
2	23854	OS	30	M	49.6@139	50.9	20/400	20/40	-3.00-1.00@115	489	Asymm.
3	27030	OS	33	M	46.8@161.7	57.2	20/400	20/160	-5.00-4.5-@135	393	Central
4	20681	OS	26	F	44.9@19	49.4	20/100	20/50	-2.00	453	Asymm.
5	30490	OS	25	F	40.3@176.7	45.1	20/400	20/40	-4.00-3.50@160	417	Totally decentered
6	11258	OD	33	F	51.3@18.7	54.3	20/60	20/60	-1.75-4.00@50	432	Asymm.
7	29176	OS	36	M	46.2@160.8	55.7	20/400	20/60	PL-9.00@145	507	Asymm.
8	32088	OD	34	F	49.6@13.5	54.5	20/200	20/100	-10.50-4.50@175	470	Central
9	32088	OS	34	F	50.3@167.5	56.4	20.400	20/60	-11.50-4.50@20	468	Central
10	30179	OS	31	M	47.3@134.4	48.8	20/30	20/25	+3.00-3.00@170	428	Asymm.
11	28499	OD	36	M	50.1@46.2	55.2	20/50	20/40	-4.25-4.00@170	444	Asymm.
12	27814	OS	21	M	46.9@138.9	51.8	20/400	20/100	-3.50-6.00@145	463	Asymm.

preoperative data including MRN ; MEDICAL RECORD NUMBER ,EYE ,AGE ,SEX ,K1 : flattest keratometry readings on pentacam, K2 : steepest keratometery readings on pentacam ,UCVA: ,uncorrected visual acuity ,BCVA: best corrected visual acuity Refract: refraction ,CCT: central corneal thickness CONE: the location of the ,cone on the pentacam

NO.	MRN	Eye	Age	Sex	K1	K2	UCVA	BCVA	Refract.	CCT	Cone
1	30490	OD	25	F	39.9@180.9	46.4	20/100	20/60	+2.25-3.00@180	443	Asymm.
2	23854	OS	30	M	41.7@160	43.3	20/30	20/30	PL-0.75@140	478	Asymm.
3	27030	OS	33	M	42.9@160.7	48.1	20/60	20/60	-1.50-6.5-@160	398	Central
4	20681	OS	26	F	40.8@09	43.6	20/40	20/25	+1.50	452	Asymm.
5	30490	OS	25	F	40.@171.71	44.6	20/40	20/28	+1.25-1.25@170	441	Totally decentered
6	11258	OD	33	F	44.7@167.1	48.9	20/60	20/40	-1.50-1.75@50	410	Asymm.
7	29176	OS	36	M	44.1@168.6	46	20/100	20/60	PL-2.50@1460	502	Asymm.
8	32088	OD	34	F	43.5@155.4	44.3	20/30	20/25	+1.50-1.50@170	461	Central
9	32088	OS	34	F	45.6@16.8	48.8	20/30	20/25	PL-1.50@170	458	Central
10	30179	OS	31	M	40.2@166	45.1	20/50	20/25	PL-1.00@170	419	Asymm.
11	28499	OD	36	M	47@46	55.3	20/400	20/100	+3.00-2.50@75	415	Asymm.
12	27814	OS	21	M	44.7@165.7	48.7	20/50	20/25	-1.00-2.75@145	456	Asymm.

postoperative data including MRN ; MEDICAL RECORD NUMBER ,EYE ,AGE ,SEX ,K1 : flattest keratometry readings on pentacam, K2 : steepest keratometery readings on pentacam ,UCVA: ,uncorrected visual acuity ,BCVA: best corrected visual acuity Refract: refraction ,CCT: central corneal thickness ,CONE: the location of the cone on the pentacam
